# Markers of Blood‐Brain Barrier Permeability and Immune Exhaustion are Present in Patients with Post‐COVID‐19 Brain Fog Compared to Controls

**DOI:** 10.1002/alz70856_103583

**Published:** 2025-12-24

**Authors:** Jennifer A. Frontera, Allal Boutajangout, Joshua Chodosh, Arjun V. Masurkar, Ludovic Debure, Alok Vedvyas, Rebecca A Betensky, Wajiha Ahmed, Mukundan Attur, Mobeena Ghuman, Manisha Bohara, Salma Hammam, Li Jiang, Jon Links, Karyn Marsh, Sujata Thawani, Daria Vasilchenko, Amin Yakubov, Yulin Ge, Thomas Wisniewski

**Affiliations:** ^1^ NYU Grossman School of Medicine, New York, NY, USA; ^2^ NYU Alzheimer's Disease Research Center, New York, NY, USA; ^3^ Center for Cognitive Neurology, New York, NY, USA; ^4^ Alzheimer's Disease Research Center, New York University Langone Health, New York, NY, USA; ^5^ Center for Cognitive Neurology, New York University Langone Health, New York, NY, USA; ^6^ NYU College of Global Public Health, New York, NY, USA; ^7^ New York University Grossman School of Medicine, New York, NY, USA; ^8^ NYU Langone Hospitals, New York, NY, USA; ^9^ Vilcek Institute of Biomedical Science, New York University School of Medicine, New York, NY, USA

## Abstract

**Background:**

While mechanisms underlying post‐COVID‐19 cognitive impairment are unknown, increased blood‐brain barrier (BBB) permeability and immune alterations have been posited as possible contributing factors.

**Methods:**

We conducted a cross‐sectional study of patients with and without prior laboratory‐confirmed COVID‐19 (COV+ vs COV‐), with no previous history of dementia/cognitive impairment. Brain fog was defined as the subjective symptoms of memory loss, confusion, and/or difficulty concentrating lasting >1 month after index SARS‐CoV‐2 infection. Fasting plasma biomarkers of inflammation (cytokines), and BBB disruption (Simoa SP‐X), as well as phosphorylated tau (pTau) were measured using Simoa HD‐X technology. Patients underwent concurrent UDS3 neuropsychiatric testing and received a formal cognitive diagnosis by a consensus group of physicians following NACC criteria. Plasma biomarker levels were compared between patients with and without brain fog, and with and without a diagnosis of MCI using Mann‐Whitney U tests.

**Results:**

We enrolled 279 patients: *N* = 51 (18%) COV‐, *N* = 228 (82%) COV+, *N* = 96 (34%) with post‐COVID brain fog, and *N* = 35 (13%) COV+ patients with newly diagnosed MCI post‐COVID. Several plasma cytokine levels (TNF‐a, IL‐4, IL‐10, IL‐22) were significantly lower in those with brain fog compared to those without (all *p* ≤0.01), while markers of BBB permeability were higher (heparin‐binding epidermal growth factor 10.1 pg/mL in brain fog patients vs 7.7 pg/mL without, *p* <0.001; vascular endothelial growth factor 72 vs. 63 pg/mL, *p* = 0.055; and placental growth factor 12.4 vs 11.4 pg/mL, *p* = 0.090). Among COV+ patients diagnosed with MCI, TNF‐a was significantly lower compared to cognitively normal subjects (1.3 vs. 1.5 pg/mL, *p* = 0.031), while pTau‐217 was significantly higher in those with MCI due to Alzheimer's (0.21 vs. 0.13 pg/mL, *p* = 0.009) compared to those without.

**Conclusions:**

Lower cytokine levels, increased markers of BBB disruption, and altered markers of tau pathology were observed in patients with brain fog and COV+ subjects with MCI, which may suggest immune exhaustion, increased BBB permeability, and disrupted tau processing as possible mechanisms of post‐COVID‐19 brain fog.

